# Clinical presentation and imaging of a rare case of Tarsal Tuberculosis

**DOI:** 10.1002/ccr3.1299

**Published:** 2018-01-17

**Authors:** Stylianos Kapetanakis, Danai Chourmouzi, Elissavet Papadopoulou, Dimitrios Oikonomou, Nikolaos Gkantsinikoudis

**Affiliations:** ^1^ Spine Department and Deformities Interbalkan European Medical Center Thessaloniki Greece

**Keywords:** Foot MRI, foot pain, foot tuberculosis, osteoarticular tuberculosis

## Abstract

A 43‐year‐old woman arrived to emergency unit of our hospital, referring intense deteriorated pain and swelling of midfoot. Rapid clinical evolvement of osteoarticular tuberculosis represents a potential clinical scenario. Clinicians should always include foot tuberculosis in differential diagnosis, in cases of severe clinical and radiological manifestations.

A 43‐year‐old woman arrived to emergency unit of our hospital, referring intense pain and swelling of midfoot. Emergence of pain was oriented 4 months ago, being initially sufficiently relieved with analgesics. However, severe deterioration and resistance in analgesic administration were recently present. Clinical examination revealed the presence of pronounced edema in mid‐ and hindfoot, severe pain in palpation, as well as restriction of mobility. Foot CT and MRI were subsequently performed, demonstrating osteitis of calcaneus, cuboid, and navicular bone. The presence of arthritis of talocalcaneal, calcaneocuboid, talonavicular, and tarsometatarsal joints was also observed (Figs 1 and 2). Differential diagnosis included primarily tarsal tuberculous osteoarthritis, neuropathic osteoarthropathy (Charcot's joint), and chronic osteomyelitis. Bone lesions biopsy with Ziehl–Neelsen staining, culture, and histopathologic examination was subsequently performed. Ziehl–Neelsen staining was negative, while culture was positive for *Mycobacterium tuberculosis* after 4 weeks. Histopathologic findings indicated tuberculosis. Mycobacteria were present in sputum examination, while imaginary characteristics of thorax CT revealed the presence of pulmonary cavitation in right lower lobe (Fig. 3), excluding thus the possibility of an alternative diagnosis. Antituberculosis regimen (isoniazid, rifampin, pyrazinamide, and ethambutol for 2 months with subsequent isoniazid and rifampin administration for 4 months) was successfully applied, confirming also the diagnosis of tarsal tuberculosis.

**Figure 1:**
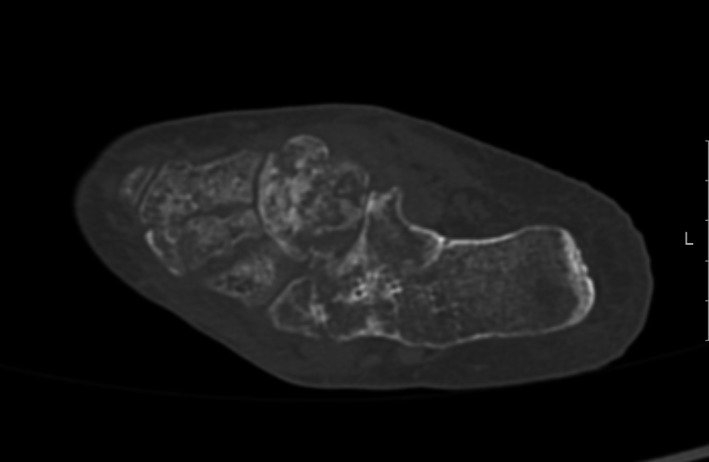
Foot CT revealed osteitis of calcaneus, cuboid, and navicular bone.

**Figure 2:**
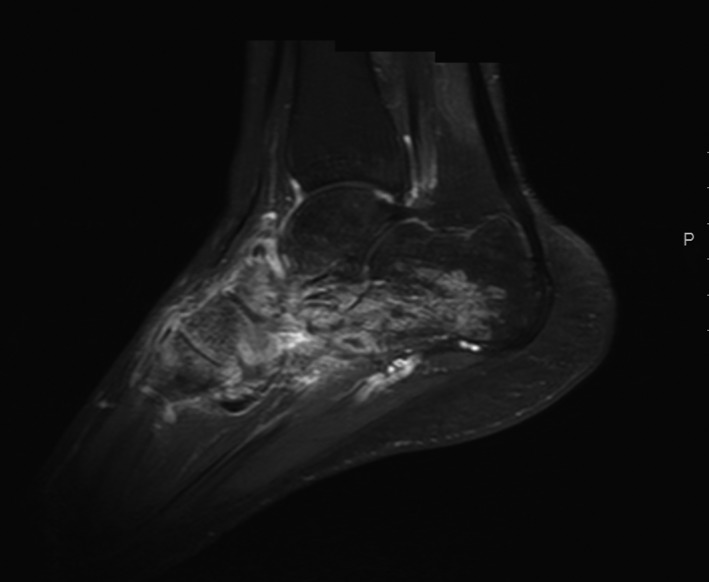
Imaginary characteristics in foot MRI indicated the presence of arthritis with disorganization of tarsal joints.

**Figure 3:**
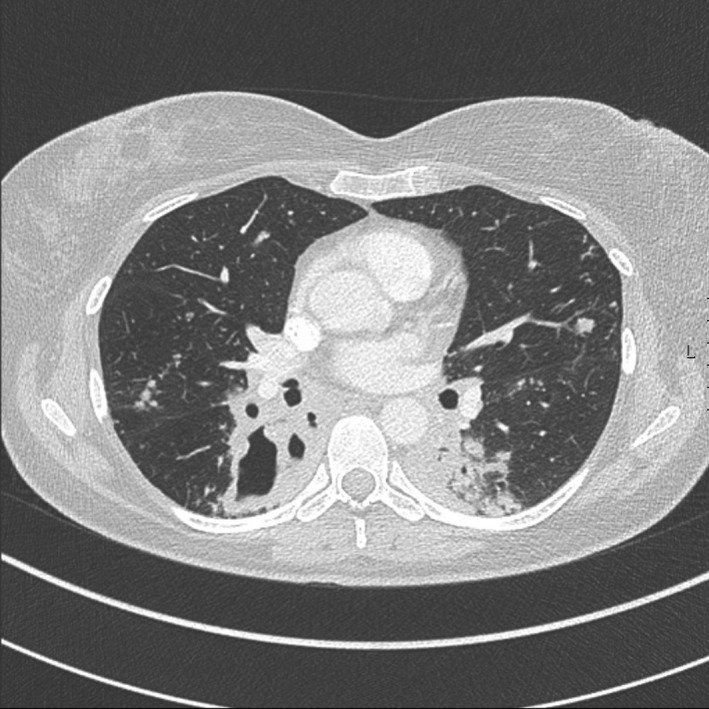
Thorax CT, lung window. Pulmonary cavitation in right lower lobe is present, demonstrating parenchymal destruction.

Foot tuberculosis is encountered in 5–10% of osteoarticular tuberculosis cases, constituting a rare clinical entity 1. Unobtrusive symptomatology with nonspecific imaginary characteristics may be responsible for delayed diagnosis 1, 2. Nevertheless, rapid clinical evolvement of osteoarticular tuberculosis represents a potential clinical scenario. Clinicians should thus always include foot tuberculosis in differential diagnosis, in cases of severe clinical and radiological manifestations.

## Authorship

SK: involved in clinical assessment, manuscript design, and general supervision; DC: involved in radiological evaluation and interpretation; EP: involved in radiological evaluation and interpretation; DO: involved in pneumonological evaluation and interpretation of submitted material; NG: involved in manuscript drafting and interpretation of submitted material.

## Conflict of Interest

None declared.
